# Salinity Stress Alters Root Morphology and Root Hair Traits in *Brassica napus*

**DOI:** 10.3390/plants8070192

**Published:** 2019-06-27

**Authors:** Mohammad Rashid Arif, M. Thoihidul Islam, Arif Hasan Khan Robin

**Affiliations:** Department of Genetics and Plant Breeding, Bangladesh Agricultural University, Mymensingh 02202, Bangladesh

**Keywords:** *Brassica napus*, reproductive growth, root hair, salinity

## Abstract

Plant roots show morphological plasticity and play a substantial role in tolerance to various edaphic stresses. The aim of this study was to explore salinity-induced morphogenic responses of root traits and root hairs of two rapeseed varieties, BARI Sarisha-8 and Binasarisha-5, at the reproductive stage and perceive the effects on their reproductive growth. The experiment was conducted in a hydroponic culture. Two treatments, 0 mM NaCl as control and 100 mM NaCl, were imposed 55 d after germination. Plants exposed to 100 mM NaCl for seven days displayed greater damage in the leaves, flowers, and siliquae compared to control. Length of root hairs on first-order and third-order lateral roots, density of root hairs on first-order lateral roots, and length of third-order lateral roots were significantly greater by 91%, 22%, 29%, and 48%, respectively, in the treated condition compared to the control. An increase in estimated root surface area by 20% under salt stress conditions indicated that the spontaneous responses of plants to uptake more water and nutrients allowed a plant to cope with stressful conditions. The results of this study suggest that any future stress breeding programs should consider plasticity of root traits intensively.

## 1. Introduction

**Figure 1:**
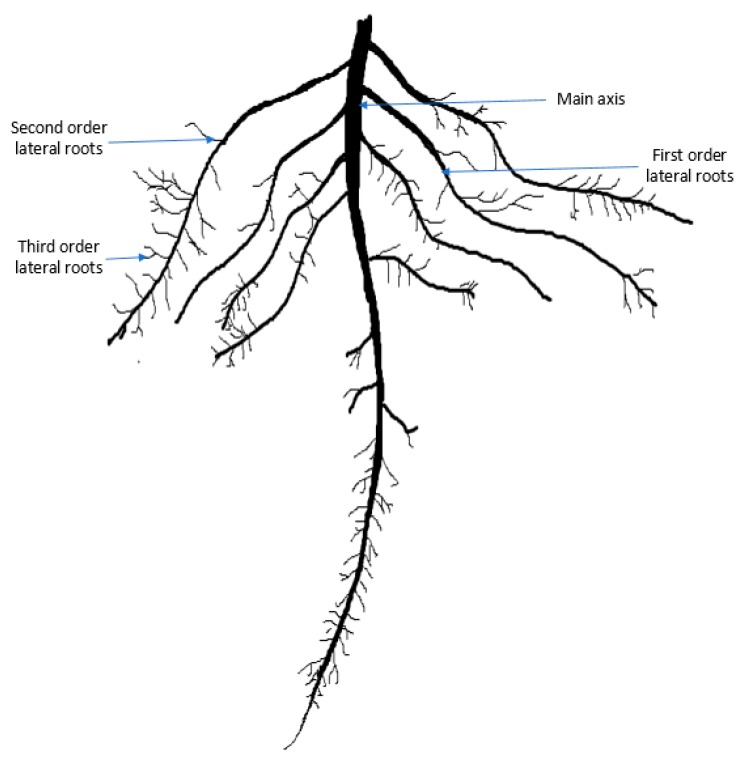
A typical root system architecture of *Brassica napus*. Root hairs appear on the main axis and all types of lateral roots.

Rapeseed mustard is the third highest source of edible oil after soybean and palm. The tetraploid *Brassica napus* L. is a dominant rapeseed species cultivated globally. In 2017–2018, 74.91 million tons of rapeseed and mustard seed were produced globally from 36.53 million hectares of land [1]. In Bangladesh, it is the top-ranked oilseed crop by a huge margin in terms of total cropped area that covers 67% of total oilseed production [2]. *Brassica napus* has a tap root system that consists of a single main root axis (embryonic roots) and lateral roots (postembryonic roots) Figure 1 [3,4,5]. The tap root is the first root to emerge from the embryo, which is anatomically well-defined [3,4,5,6,7]. Lateral roots are postembryonically formed, initiated from existing roots, and are generally branched [4,5,7,8]. Root hairs are unicellular, tubular projections from the modified epidermal cells of the root that increase the surface area of roots for nutrient and water uptake [9].

Salinity generates adverse environmental and hydrological conditions that restricts regular crop production. Salt stress affects seed germination, seedling establishment, growth, and development of plants by altering physiological and metabolic processes that eventually lead to reduction in yield [10]. Salinity reduces leaf area and photosynthetic rate [11,12] and alters the light phase of photosynthesis [13,14] by inducing osmotic stress. Physiological alteration under salinity stress includes nutritional imbalance and low soil water potential because of the excess accumulation of Na^+^ and Cl^–^ ions [15]. Ion toxicity inhibits the enzymatic function of vital biological processes [16]. Salinity affects the development of male and female reproductive organs of plants, which are very sensitive to stress, decreases plant fecundity [17], and, thus, directly affects the yield of plants.

In *Brassica* species, salinity stress was reported to reduce growth, seed yield, and oil production by exhibiting, to a great extent, interspecific variation in salinity tolerance [18]. The general negative impact of salinity stress on *Brassica* crops reported earlier includes reduction in plant height, size and yield, and deterioration of seed quality [19]. Several reports on salt stress in *Brassica* species stated a decline of the shoot/root ratio [20,21,22], although there are also contradictory indications [23].

The plasticity of roots under salinity stress is the key to coping with stressful conditions, as root surfaces first get exposed to environmental stress [24]. Root morphological plasticity may include preventing accumulation of salt in roots so that water uptake may continue from saline soils [25]. Salt stress largely regulates formation and development of root hairs [9]. Epidermal development of roots shows plasticity, as external factors modulate epidermal cell types and initiation of root hairs [9,26]. Plasticity in the development of the root epidermis as a response to varying environmental conditions might indicate a function of root hairs in sensing environmental signals upon which plants adapt in stress conditions [27,28,29,30,31,32]. Salt stress at a lower concentration (5 g L^−1^ NaCl) induced abundant root hairs, but with greater salt concentrations (10 and 15 g L^−1^ NaCl) gradually lower numbers of root hairs were counted [33]. Density of root hair population, length, and diameter of individual root hairs largely determines total root surface area in plants. In stressful environments, either the determinants of root surface area or the root surface area, per se, were found to be less in both monocots (e.g., wheat and barley [34,35,36]) and in dicots (e.g., *Arabidopsis* [9] and mulberry [37]). Under salinity stress in particular, both root hair length and density of root hairs per unit surface area were less than 25% and 40% compared to those of untreated wheat genotypes grown hydroponically [36]. By contrast, in the case of seedlings of *Silene vulgaris*, two determinants of total root surface area—total root length and branching density—increased under moderate drought stress [38].

A thorough understanding of root system architecture with a special emphasis on root hair traits under salinity stress would be helpful for future rapeseed breeding. This study was planned to explore the effects of salinity stress at the reproductive stage. The focus of the experiment was on determining the alteration in root morphological traits and root hair traits, with a special emphasis on fine roots, and scoring the effect of salt stress on reproductive organs.

## 2. Materials and Methods

### 2.1. Plant Culture and Management

The experiment was carried out in a growth chamber at the Department of Genetics and Plant Breeding, Bangladesh Agricultural University, Mymensingh, Bangladesh. Two rapeseed (*Brassica napus*) cultivars, BARI Sarisha-8 and Binasarisha-5, were used in this study. Seeds of BARI Sarisha-8 were collected from Bangladesh Agricultural Research Institute, and those of Binasarisha-5 were collected from Bangladesh Institute of Nuclear Agriculture (BINA), Mymensingh. Seeds were germinated on one layer of moistened filter paper in Petri dishes. One week after germination, uniform, healthy, dark green seedlings were transferred to a hydroponic system. A modified Hoagland solution was used to supply suitable amounts of nutrients to the plants [39]. Plants were organized following a completely randomized design with two treatments and four replicates per treatment for each variety. The composition and concentration of the nutrients were 1 mM NH_4_NO_3_, 0.6 mM NaH_2_PO_4_.H_2_O, 0.6 mM MgCl_2_.H_2_O, 0.3 mM K_2_SO_4_, 0.3 mM CaCl_2_.H_2_O, 50 μM H_3_BO_3_, 90 μM Fe-EDTA, 9 μM MnSO_4_.4H_2_O, 0.7 μM ZnSO_4_.7H2O, 0.3 μM CuSO_4_.5H_2_O, and 0.1 μM NaMoO_4_.2H_2_O dissolved in water [35,36]. The nutrient solution was restored once a week. 

Periodically, the trays with plants were rotated to change their position randomly to avoid positional effect. All plants were managed in a plant culture room under the same environmental conditions. The pH of the nutrient solution was maintained between 5.8 and 6.0. A 16:8 h day:night ratio was maintained in the plant culture room. The temperature of the culture room was maintained at 20 ± 2 °C. 

### 2.2. Salinity Treatment, Injury Scoring, and Data Collection

**Figure 2:**
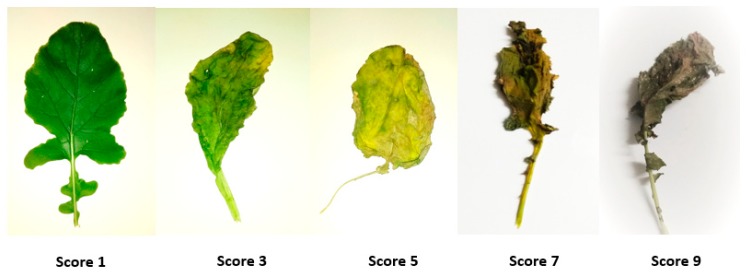
Leaf injury scoring for salinity stress.

**Figure 3:**
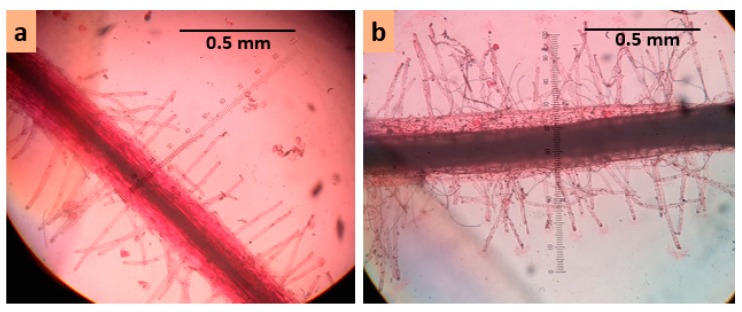
Measurement of root hair traits under microscope (**a**) first-order lateral root of Binasarisha-5 in control conditions, and (**b**) first-order lateral root of BARI sarisha-8 in 100 mM NaCl treated conditions.

At day 55 (flowering stage), two treatments, 0 mM and 100 mM NaCl, were imposed in order to induce salt stress. A salinity level of 100 mM concentration increased electric conductivity of the growth medium by about 10–11 dS m^–1^. Salt injury symptoms were assessed for different plant parts such as leaves, flowers, and siliqua at three, five, and seven days after the treatment imposition. Visible salt injury was scored using a 1–9 scale (see Table 1, Figure 2). Plants were destructively harvested at seven days after the commencement of salt treatment to collect data on root and root hair traits. Root traits like length and diameter of main root axis; length, diameter, and density of first-, second-, and third-order lateral roots; and root hair traits like length, diameter, and density of root hair of first-, second-, and third-order lateral roots were measured. Length of the main axis and first-order lateral roots were measured by a centimeter ruler. All other traits were measured under a light microscope at 100× magnification using a micrometer scale (Figure 3). Acetocarmine solution of 0.5% prepared with 45% glacial acetic acid was used to make roots and root hairs clearly visible under a light microscope.

### 2.3. Estimation of Root Surface Area

Root surface area was calculated by assuming that roots and root hairs were spherical in cross section. Thus, it could be calculated with the equation of a cylinder [35,36]. The equation was as follows:

Estimated root surface area = πD_m_L_m_ (1+ a_1_n_1_πD_1_L_1_ (1 + a_rh1_n_rh1_πD_rh1_L_rh1_ + a_2_n_2_πD_2_L_2_ (1 + a_rh2_n_rh2_πD_rh2_L_rh2_ + a_3_n_3_πD_3_L_3_ (1 + a_rh3_n_rh3_πD_rh3_L_rh3_)))) (1)

where, D_m_ and L_m_ are the diameter and length of main axis, respectively; D_i_ and L_i_ are the diameter and length of the *i*th order lateral root, respectively; D_rhi_ and L_rhi_ are the diameter and length of the root hair at the *i*th order lateral root, respectively; a_i_ is the proportion of the length of roots that bear *i*th order lateral roots; a_rhi_ is the proportion of the length of *i*th order lateral roots that bear root hairs; n_i_ is the density of *i*th order lateral roots; and n_rhi_ is the density of the root hair at *i*th order lateral roots.

Details of Equation (1) are given in Supplementary Appendix 1. 

### 2.4. Statistical Analysis

Minitab 17 statistical software package (Minitab Inc., State College, PA, USA) was used for data analysis. Analysis of variance (ANOVA) was performed following a generalized linear model to reveal deviations among treatments, varieties, and treatment × varieties. A post hoc analysis was conducted following Tukey’s pairwise comparisons to separate means of treatment, variety, and treatment x variety interactions. A principal component analysis (PCA) was carried out for selected traits that showed significant variations to discover complex patterns in the data and associations among measured traits. ANOVA of the PC scores was performed for treatment × variety interactions following a similar generalized linear model. A Pearson correlation analysis was carried out using Minitab 17 statistical software package for selected traits to explore relationships among them. 

## 3. Results 

### 3.1. Effects of Salt Treatment

**Figure 4:**
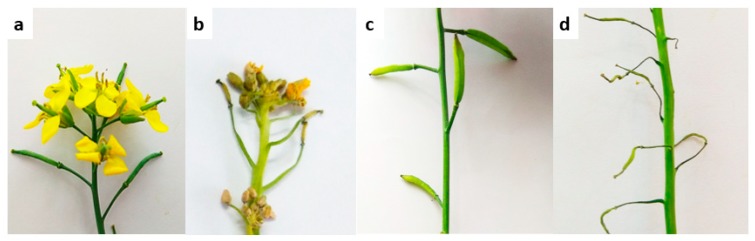
Effect of salinity on the reproductive organs at 7 d after treatment. (**a**) Inflorescence under control conditions; (**b**) inflorescence under 100 mM NaCl; (**c**) siliquae at control conditions; and (**d**) siliquae under 100 mM NaCl.

**Figure 5:**
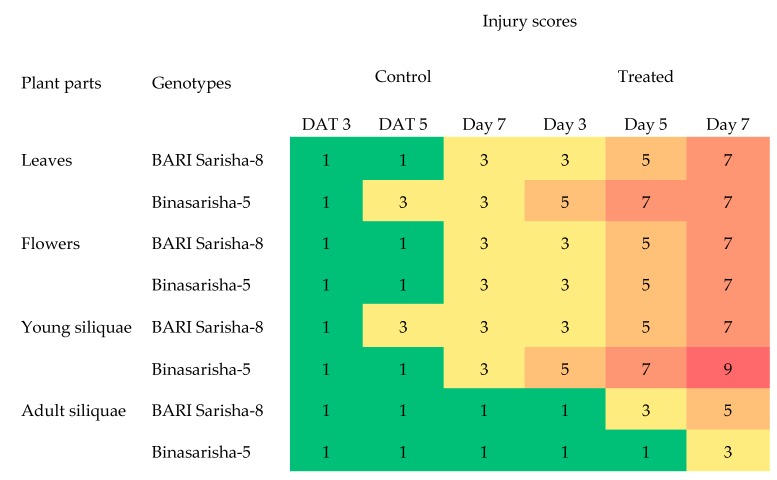
Injury scoring of two rapeseed genotypes, under control and 100 mM NaCl treated conditions at the reproductive stage, in a heat map created using conditional formatting in Microsoft Excel (Supplementary Datasheet 1). DAT, days after treatment. Each data point is the median value of five independent observations. Green, yellow, and red colors represent no, moderate, and severe injury levels.

Salt treatment caused visible symptoms on leaves, flowers, and siliquae. At two days after treatment application, leaves and flowers started to discolor and roll. At seven days after treatment, most leaves became nearly dead, new buds did not open, and young siliquae were completely dead. Adult siliquae still had their growth, but they were affected too, as they started to slightly discolor, wrinkle, and showed signs of early maturation (Figure 4). Salinity damage increased gradually from day 3 to day 7 after treatment in treated plants (Figure 5). Flowers were almost similarly affected for both genotypes; however, the leaves and young siliquae were more affected in Binasarisha-5, whereas adult siliquae were more affected in BARI Sarisha-8.

**Figure 6:**
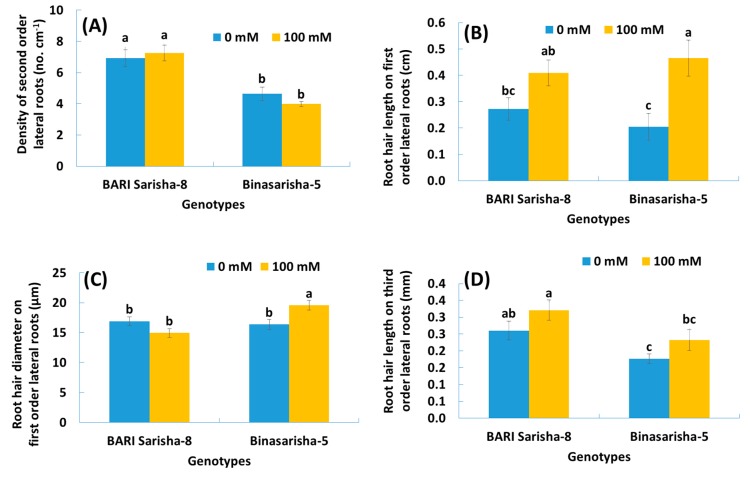
(**A**) Density of second-order lateral roots, (**B**) length of root hairs originating on first-order lateral roots, (**C**) diameter of root hairs originating on first-order lateral roots, and (**D**) length of root hairs originating on third-order lateral roots of two rapeseed varieties under 0 mM and 100 mM NaCl treatments. Vertical bars indicate standard error of mean of four replicates against each variable. Different letters indicate significant differences among the genotype × treatment interactions.

**Figure 7:**
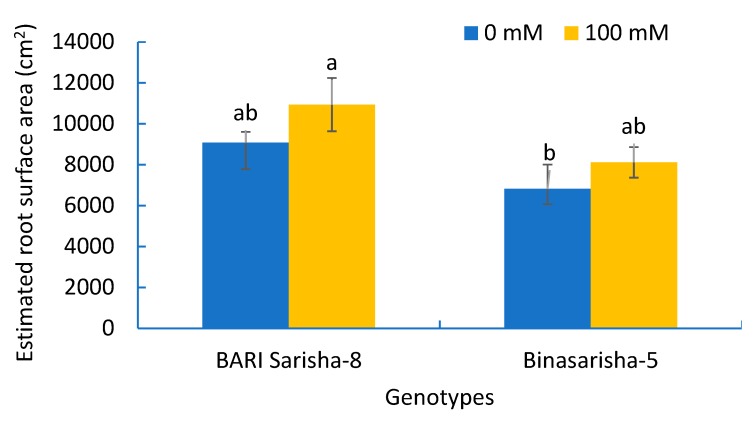
Estimated root surface area of two rapeseed varieties under 0 mM and 100 mM NaCl treatments. Vertical bars indicate standard error of mean of four replicates against each variable. Different letters indicate significant differences among the genotype × treatment interactions.

A significant effect of salt stress was found in root hair length at seven days after salinity treatment (Table 2). Root hair length on first-order lateral roots increased by 91% (*P* < 0.001) (Figure 6B), and root hair length on third-order lateral roots increased by 22% (*P* < 0.05) (Figure 6D) under salinity treatment compared to control. In addition, the length of third-order lateral roots increased by 48% (Figure S2), density of root hair on first-order lateral roots increased by 29% (*P* < 0.1) (Figure S5), and estimated root surface area increased by 20% (*P* < 0.1) (Figure 7), whereas the diameter of root hairs of the third-order lateral roots decreased by 9% (*P* < 0.1) (Figure S3).

### 3.2. Varietal Differences

It was observed that different root and root hair traits were significantly different in two varieties (Table 2). BARI Sarisha-8 accounted for significantly greater first-order lateral root diameter (Figure S1), second-order lateral root density (Figure 6A), third-order lateral root density (Figure S4), density of root hairs on first-order lateral roots (Figure S5), length of root hairs on third-order lateral roots (Figure 6D), and estimated root surface area (Figure 7) compared to Binasarisha-5. On the other hand, Binasarisha-5 accounted for a greater value of third-order lateral root diameter (Figure S3) and diameter of root hairs on the first-order lateral roots (Figure 6C).

### 3.3. Treatment × Variety Differences

Traits like the diameter of first-order lateral roots (P < 0.05) (Figure S1), density of third-order lateral roots (*P* < 0.05) (Figure S4), and diameter of root hair of first order lateral roots (*P* < 0.01) (Figure 6C) showed a significantly different salinity treatment × variety interaction (Table 2).

### 3.4. Trait Associations 

**Figure 8:**
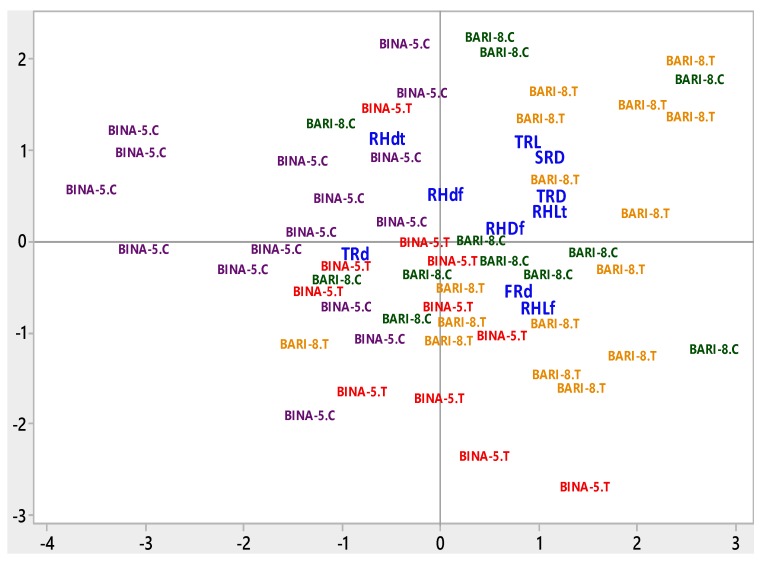
Biplot of root and root hair traits. BARI-8.C = BARI Sarisha-8 under Control, BARI-8.T = BARI Sarisha-8 under Treated condition, BINA-5.C = Binasarisha-5 Control, BINA-5.T = Binasarisha-5 under Treated condition, FRd = Diameter of first-order lateral roots, SRD = Density of second-order lateral roots, TRL = Length of third-order lateral roots, TRd = Diameter of third-order lateral roots, TRD = Density of third-order lateral roots, RHLf = Length of root hairs on first-order lateral roots, RHdf = Diameter of root hairs on first-order lateral roots, RHDf = Density of root hairs on first-order lateral roots, RHLt = Length of root hairs on third-order lateral roots, and RHdt = Diameter of root hairs on third-order lateral roots.

The most apposite combination of the studied traits was obtained from the principal component analysis where the vector length on biplot exhibited the magnitude of variation explained by respective trait and variety-treatment combinations in the PCA (Figure 8). The first four principal components (PC) explained 62.4% of the total data variation for the effect of salinity stress on some important root and root hair traits. PC1, PC2, PC3, and PC4 explained 23.1%, 14.7%, 13.4%, and 11.1% data variation respectively (Table 3). PC1 accounted for a greater separation of Binasarisha-5 under control treatment from other interactions for a larger diameter of laterals and root hairs on third-order branches (Figure 8). 

For measuring the mutual relationship among different root and root hair traits, correlation analyses were conducted. Length of third-order lateral roots was positively associated with the density of second-order lateral roots, and density of third-order lateral roots was negatively associated with the diameter of third-order lateral roots. The density of third-order lateral roots was also positively correlated with the length and density of second-order lateral roots, whereas it negatively correlated with the diameter of third-order lateral roots. The length of root hairs on first-order lateral roots was positively linked with the density of root hairs on first-order lateral roots and the length and density of root hairs on third-order lateral roots. The densities of root hairs on first- and third-order lateral roots were positively correlated (Table 4). 

## 4. Discussion

### 4.1. Effects of Salinity Stress on Shoot and Root Morphologies

Under salt-treated conditions, salt injury scores reflected severity of damage to the leaf, flower and siliqua (Figure 4 and Figure 5). It is likely that drying of leaves under salinity stress is caused by excessive accumulation of the salt that enhanced leaf senescence [40]. In addition, degradation of chlorophyll molecules under salinity stress may have caused discoloration of leaves [41]. Similar results were reported in wheat and barley [42], tomato [43], *Prosopis alba* [44], and maize [45]. Wilting and drying of reproductive parts (i.e., flowers and fruits) were reported in canola [17] and peas [46]. These detrimental effects of salinity on plants were directed largely through osmotic stress, ion toxicity, and mineral deficiency [47,48,49,50]. 

Root system architecture and expansion is mostly regulated by water and nutrient uptake efficiency; however, these root processes are affected differently by excess salinity [51]. Plants use their root plasticity to survive in stress conditions [52,53]. An increase in the length of the third-order lateral root (Figure S2) in this study highlights root system plasticity under salt stress. In previous studies, a few contradictory results were reported related to root elongation. In a number of crops, such as rice, wheat, and *Arabidopsis,* the rate of root elongation decreased under salinity stress [54,55,56]. Again, in the cases of *Arabidopsis thaliana* [57] and *Silene vulgaris* [38], lateral roots were found to show promoted elongation. Elongation of roots is the result of cell division and cell expansion in the root apical meristem. We can assume that salinity may alter root elongation both by promoting and reducing cell division and expansion [58].

Root hairs contribute largely to root surface area [35]. In our study, length and density of root hairs as well as root surface area increased in the treated plants, whereas diameter decreased (Figure 6B; Figures S5–S7). Bates and Lynch [59] reported in *Arabidopsis thaliana* that low phosphorus availability increased root hair length and, thus, provided an opportunity to attain more nutrition. Robin et al. [36] found that root hair length and density in wheat were reduced after 12 days of salinity stress. In earlier reports, the root variables that contributed to root surface area were found to be reduced under stressed conditions [9,34,35,36,37]. Considering that root surface area is correlated with nutrient uptake potential, our results suggest that the *Brassica napus* genotypes, at the reproductive stage, increased their nutrient uptake potential as an adaptive mechanism under salinity stress. In this study, the leaves and young siliquae of the Binasarisha-5 variety were severely injured at day 5 and day 7 under treated conditions compared to control (Figure 5). At the same time, length of third-order lateral roots as well as length and diameter of root hairs of first-order lateral roots significantly increased (Figures S2 and S5). These results suggest that when shoot parts become severely injured, the Binasarisha-5 variety increases finer root production to increase the overall absorption area as an adaptive mechanism of stress tolerance and as a genotype-specific response.

### 4.2. Varietal Variations 

Significant variations in root morphology between two rapeseed varieties (Table 2; Figure 6) indicated inter-cultivar genetic potentials [60,61,62]. Investigating the inter-cultivar genetic variation for salinity tolerance is important to improve their tolerance levels to abiotic stress [63]. Our study exposed the differences in magnitude of salinity effects between two varieties. Varietal differences in response to salinity depends on hereditary differences in morphological plasticity and physiological deviation [40]. 

Root interaction with a changing environment is a complex phenomenon that differs among genotypes and intensity of stress [26]. Identifying and understanding the pattern of genotype × environment is imperative in order to be able to conduct efficient genetic manipulations [7]. For that, different genotypes may respond contrarily under stressful conditions and show different magnitudes of tolerance or susceptibility to stress. Significant treatment × variety interactions for root and root hair traits in our study (Table 2; Figure 6C; Figures S1 and S4) indicated that the genotypes reacted differently under salinity stress. The trait-specific variation in salinity tolerance could be exploited in breeding for salinity tolerance through pyramiding tolerance traits. 

### 4.3. Trait Associations

Root elongation and branching are the progressive developmental processes of root traits [5,64,65]. In root systems, divergence in the attributes for elongation and branching create morphological differences in length, diameter, and density of different-order roots [5,66,67]. Thus, the presence of a relationship among those traits is inevitable. However, a wide range of inter- and intra-specific variation is expected. Principal component analysis and correlation analysis revealed the relationship among the root and root hair traits in our study (Table 3 and Table 4; Figure 8). For example, length and density of root hairs had positive associations between them and had negative associations with the diameter of root hairs. The diameter and density of third-order lateral roots had a negative relationship, but the relationship for these two traits of root hairs remains uncertain, with contradictory results found in other studies even within the same species [68,69,70]. The relationship among the root and root hair traits obtained from the experiment gives novel insight into root and root hair trait associations and provides facts that can be exploited in further studies.

## 5. Conclusions

Salinity is one of the most injurious abiotic stresses for plants that alters different morphological and physiological traits of plants to an abnormal state. The *Brassica napus* genotypes responded broadly in terms of their physio-morphological features and differential tolerance to salinity stress. Our study found that salt stress significantly alters root hair traits as well as estimated root surface area, which in turn may represent a strategy to confront salt stress. The results of this study can be utilized to develop plants with improved root system architecture to acclimate to stressful conditions. 

## Figures and Tables

**Table 1:** Criteria for scoring visual salt injury in rapeseed at the reproductive stage.

Score	Leaves	Flowers	Siliquae
1	Normal color and growth	Healthy and of normal color, blossoming	Normal color and growth
3	Nearly normal conditions, but leaf tip discoloration and wilting have started	Bud does not blossom properly, opened bud has started shrinking	Nearly normal, but slight discoloration has started
5	The leaf has rolled, most of the leaf has discolored and started to dry	Petal compacted or twisted; young bud has started to die instead of blossoming	No further growth or very slow growth, discolored
7	The leaf is mostly dry and totally discolored	unopened flower bud has died, open flower has dried	Growth totally ceased, drying
9	The leaf is dead or near death	Most of the bud is dead or near death	Siliquae dead or near death

**Table 2:** Mean squares of the respective sources of variances with significance levels under salinity stress for lateral root and root hair traits.

Source of Variation	df	Lateral Root Traits				Root Hair Traits			
		First Order	Second Order	Third Order		On First Order			On Third Order
		Diameter	Density	Diameter	Density	Length	Diameter	Density	Length
Treatments (T)	1	0.08	0.47	0.0006	0.75	0.58 ***	5.39	330.31	0.05 *
Varieties (V)	1	0.14 *	123.49 ***	0.0165 **	4.57 *	0.00	61.56 *	535.39 *	0.11 **
T × V	1	0.17 *	3.75	0.0024	4.27 *	0.06	98.56 **	0.16	0.00
Error	60	0.03	2.99	0.0022	1.03	0.05	9.14	130.96	0.01

*, **, and *** = Significant at ≤ 5%, ≤ 1%, and ≤ 0.1% levels of probability, respectively.

**Table 3:** Coefficients of principal components (PCs) and mean PC scores of each genotype × treatment combination. Different letters indicate significant differences among the genotype × treatment interactions after Tukey’s pairwise comparisons.

Variable			PC1	PC2	PC3	PC4
Lateral roots	1st order	Diameter	0.284	−0.354	0.504	0.225
	2nd order	Density	0.424	0.194	0.16	0.125
	3rd order	Length	0.36	0.493	−0.114	−0.112
	3rd order	Diameter	−0.289	−0.172	0.224	−0.286
	3rd order	Density	0.416	0.082	−0.131	0.257
Root hairs on	1st order	Length	0.3	−0.456	−0.048	−0.502
	1st order	Diameter	0.057	0.119	0.381	−0.546
	1st order	Density	0.283	−0.068	−0.55	−0.392
	3rd order	Length	0.405	−0.044	0.331	−0.018
	3rd order	Diameter	−0.131	0.574	0.283	−0.262
% Variation explained			23.1	14.7	13.4	11.1
*P*-value			< 0.001	4.9	41.2	19.2
Mean PC scores with standard error						
BARI Sarisha-8. Control			0.61 ± 0.36 ab	0.35 ± 0.34 a	0.3 ± 0.46 a	0.23 ± 0.18 ab
Binasarisha-5. Control			−1.55 ± 0.28 c	0.32 ± 0.25 a	−0.05 ± 0.16 a	−0.02 ± 0.26 ab
BARI Sarisha-8. Treated			1.17 ± 0.27 ab	0.003 ± 0.31 ab	−0.36 ± 0.21 a	0.25 ± 0.34 ab
Binasarisha-5. Treated			−0.11 ± 0.24 b	−0.86 ± 0.36 b	0.27 ± 0.47 a	−0.58 ± 0.23 b

**Table 4:** Pearson correlation coefficients among root and root hair traits.

			Lateral Root Traits						Root Hair Traits				
			First Order	Second Order		Third Order			On First Order			On Third Order	
			Diameter	Length	Density	Length	Diameter	Density	Length	Diameter	Density	Length	Diameter
**Lateral**	**2nd order**	**Length**	0.20										
	**2nd order**	**Density**	0.23	0.13									
	**3rd order**	**Length**	0.02	−0.15	0.39 **								
	**3rd order**	**Diameter**	−0.06	−0.03	−0.14	−0.30 *							
	**3rd order**	**Density**	0.20	0.37 **	0.31 *	0.35 **	−0.27 *						
**Root hairs on**	**1st order**	**Length**	0.23	0.20	0.05	0.02	0.00	0.11					
	**1st order**	**Diameter**	0.09	−0.22	−0.12	0.10	−0.02	−0.02	0.15				
	**1st order**	**Density**	−0.14	−0.09	0.11	0.23	−0.12	0.20	0.35 **	−0.08			
	**3rd order**	**Length**	0.32 *	0.01	0.32 *	0.09	−0.15	0.14	0.27 *	0.01	0.09		
	**3rd order**	**Diameter**	−0.23	−0.39 **	0.02	0.08	0.15	−0.15	−0.25	0.13	−0.15	0.12	
	**3rd order**	**Density**	0.24	0.12	0.11	0.09	−0.24	0.24	0.27 *	0.25	0.32 *	0.18	−0.18

* and ** = Significant at ≤ 5% and ≤ 1% levels of probability, respectively.

## Supplementary Materials

The following are available online at https://www.mdpi.com/2223-7747/8/7/192/s1.
