# Supplementary material for: Salinity Stress Alters Root Morphology and Root Hair Traits in Brassica napus
Source: Plants (Basel). 2019 Jun 27;8(7):192. doi: 10.3390/plants8070192 (PMC6681291; doi:10.3390/plants8070192)
Supplement: Supplementary file 1 [file plants-08-00192-s001.zip › plants-517602-supplementary-v2/Supplemental Data.docx]

**Supplemental data**

**Figure S1.** Diameter of first order lateral roots of two rapeseed varieties under 0 mM and 100 mM NaCl treatments. Vertical bars indicate standard error of mean of four replicates against each variable. Different letters indicate significant difference among the genotype × treatment interactions.

**Figure S2.** Length of third order lateral roots of two rapeseed varieties under 0 mM and 100 mM NaCl treatments. Vertical bars indicate standard error of mean of four replicates against each variable. Different letters indicate significant difference among the genotype × treatment interactions.

**Figure S3.** Diameter of third order lateral roots of two rapeseed varieties under 0 mM and 100 mM NaCl treatments. Vertical bars indicate standard error of mean of four replicates against each variable. Different letters indicate significant difference among the genotype × treatment interactions.

**Figure S4.** Density of third order lateral roots of two rapeseed varieties under 0 mM and 100 mM NaCl treatments. Vertical bars indicate standard error of mean of four replicates against each variable. Different letters indicate significant difference among the genotype × treatment interactions.

**Figure S5.** Density of root hairs originated at first order lateral roots of two rapeseed varieties under 0 mM and 100 mM NaCl treatments. Vertical bars indicate standard error of mean of four replicates against each variable. Different letters indicate significant difference among the genotype × treatment interactions.

**Figure S6.**  Diameter of root hair originated at third order lateral roots of two rapeseed varieties under 0 mM and 100 mM NaCl treatments. Vertical bars indicate standard error of mean of four replicates against each variable. Different letters indicate significant difference among the genotype × treatment interactions.

**Supplementary Appendix 1.** Breakdown of equation 1 for estimating the potential root surface area of component roots.

Estimated surface area of main root axis = πD_m_L_m_
1^st^ order= axes + root hairs = πD_m_L_m_a_1_n_1_πD_1_L_1_ + πD_m_L_m_a_1_n_1_πD_1_L_1_ a_rh1_n_rh1_πD_rh1_L_rh1_
2^nd^ order= axes + root hairs = πD_m_L_m_a_1_n_1_πD_1_L_1_ a_2_n_2_πD_2_L_2_ + πD_m_L_m_a_1_n_1_πD_1_L_1_ a_2_n_2_πD_2_L_2_ a_rh2_n_rh2_πD_rh2_L_rh2_
3^rd^ order= axes + root hairs = πD_m_L_m_a_1_n_1_πD_1_L_1_ a_2_n_2_πD_2_L_2_ a_3_n_3_πD_3_L_3_ + πD_m_L_m_a_1_n_1_πD_1_L_1_ a_2_n_2_πD_2_L_2_ a_3_n_3_πD_3_L_3_a_rh3_n_rh3_πD_rh3_L_rh3_

Here,
D_m_ and L_m_ are the diameter and length of main axis respectively.
D_i_ and L_i_ are the diameter and length of ith order lateral root respectively.
D_rhi_ and L_rhi_ are the diameter and length of root hair at ith order lateral root respectively.
a_i_ is the proportion of the length of roots that bear ith order lateral roots.
a_rhi_ is the proportion of the length of ith order lateral roots on that bears root hairs.
n_i_ is the density of ith order lateral roots.
n_rhi_ is the density of root hair at ith order lateral roots.

**Estimated root surface area**

= πD_m_L_m_ + πD_m_L_m_ a_1_n_1_πD_1_L_1_ + πD_m_L_m_ a_1_n_1_πD_1_L_1_ a_rh1_n_rh1_πD_rh1_L_rh1_ + πD_m_L_m_ a_1_n_1_πD_1_L_1_ a_2_n_2_πD_2_L_2_ + πD_m_L_m_ a_1_n_1_πD_1_L_1_ a_2_n_2_πD_2_L_2_ a_rh2_n_rh2_πD_rh2_L_rh2_ + πD_m_L_m_ a_1_n_1_πD_1_L_1_ a_2_n_2_πD_2_L_2_ a_3_n_3_πD_3_L_3_ + πD_m_L_m_ a_1_n_1_πD_1_L_1_ a_2_n_2_πD_2_L_2_ a_3_n_3_πD_3_L_3_ a_rh3_n_rh3_πD_rh3_L_rh3_

= πD_m_L_m_ (1+ a_1_n_1_πD_1_L_1_ + a_1_n_1_πD_1_L_1_ a_rh1_n_rh1_πD_rh1_L_rh1_ + a_1_n_1_πD_1_L_1_ a_2_n_2_πD_2_L_2_ + a_1_n_1_πD_1_L_1_ a_2_n_2_πD_2_L_2_ a_rh2_n_rh2_πD_rh2_L_rh2_ + a_1_n_1_πD_1_L_1_ a_2_n_2_πD_2_L_2_a_3_n_3_πD_3_L_3_ + a_1_n_1_πD_1_L_1_ a_2_n_2_πD_2_L_2_ a_3_n_3_πD_3_L_3_ a_rh3_n_rh3_πD_rh3_L_rh3_)

= πD_m_L_m_ (1+ a_1_n_1_πD_1_L_1_ (1 + a_rh1_n_rh1_πD_rh1_L_rh1_ + a_2_n_2_πD_2_L_2_ + a_2_n_2_πD_2_L_2_ a_rh2_n_rh2_πD_rh2_L_rh2_+ a_2_n_2_πD_2_L_2_ a_3_n_3_πD_3_L_3_ + a_2_n_2_πD_2_L_2_ a_3_n_3_πD_3_L_3_ a_rh3_n_rh3_πD_rh3_L_rh3_))

= πD_m_L_m_ (1+ a_1_n_1_πD_1_L_1_ (1 + a_rh1_n_rh1_πD_rh1_L_rh1_ + a_2_n_2_πD_2_L_2_ (1 + a_rh2_n_rh2_πD_rh2_L_rh2_+ a_3_n_3_πD_3_L_3_ + a_3_n_3_πD_3_L_3_ a_rh3_n_rh3_πD_rh3_L_rh3_)))

= πD_m_L_m_ (1+ a_1_n_1_πD_1_L_1_(1 + a_rh1_n_rh1_πD_rh1_L_rh1_ + a_2_n_2_πD_2_L_2_ (1 + a_rh2_n_rh2_πD_rh2_L_rh2_+ a_3_n_3_πD_3_L_3_ (1 + a_rh3_n_rh3_πD_rh3_L_rh3_))))
